# Mesh Migration Into the Sigmoid Colon Identified After Positive Fecal Occult Blood Test: A Case Report

**DOI:** 10.7759/cureus.106931

**Published:** 2026-04-13

**Authors:** Caoimhin J McDermott, Andrew Coveney

**Affiliations:** 1 Department of General Surgery, Sir Charles Gairdner Hospital, Perth, AUS; 2 Curtin Medical School, Curtin University, Perth, AUS

**Keywords:** case report, inguinal hernia complications, inguinal mesh complications, laparoscopic colorectal surgery, mesh erosion, open inguinal hernia repair, surgical case reports

## Abstract

Inguinal hernia repair with mesh reconstruction is amongst the most frequently performed elective general surgical procedures and is associated with well-recognized complications such as recurrence, infection, and chronic pain. Rarer complications of mesh repair have also been reported, particularly since the advent of laparoscopic inguinal hernia repair. This report highlights an important, rare complication of left-sided hernia repair, which should be considered by surgeons, particularly in the presence of certain associated risk factors.

A 72-year-old man underwent colonoscopy following a positive fecal occult blood test (FOBT). The patient had a history of open left inguinal hernia repair 19 years previously, complicated by chronic wound infection. Computed tomography (CT) showed three nonspecific calcified fragments in the proximal sigmoid colon with associated diverticulosis. Flexible sigmoidoscopy confirmed erosion of the surgical mesh through the colonic mucosa. The patient underwent a laparoscopic high anterior resection with primary anastomosis and made an unremarkable recovery.

This case highlights a rare but important late complication of a common elective general surgical procedure and adds to the body of evidence in this area. Sigmoid diverticulosis may represent a rare complicating factor in elective left-sided hernia repair.

## Introduction

Inguinal hernia repair with mesh reconstruction is one of the most common elective general surgical procedures with well-recognized associated complications, including recurrence, infection, and chronic pain [1,2]. Less common complications of hernial mesh repair have also been described, particularly since the advent of pre-peritoneal laparoscopic inguinal hernia repair [3].

A recent meta-analysis comparing common complications associated with inguinal hernia repair with mesh reconstruction found no significant difference in the incidence of hernia recurrence and postoperative seroma between the three most common types of inguinal hernia repair, that is, the open Lichtenstein technique, the laparoscopic preperitoneal repair (TAPP), and the totally extraperitoneal repair (TEP). The incidence of hematoma, wound infection, and chronic pain was significantly reduced in hernias repaired via minimally invasive technique (TAPP and TEP) compared to the open Lichtenstein repair [4].

This report discusses a case involving mesh erosion into the sigmoid colon following a previous open left inguinal hernia repair with a polypropylene mesh plug. Given the rarity of such complications, the true incidence is unknown. However, a review of cases reported found that visceral complications were reported more commonly in those who had a previous laparoscopic inguinal hernia repair, particularly TAPP [5].

The patient was managed in a high-volume colorectal and general surgical unit in a tertiary referral centre in Perth, Western Australia, consisting of five subspecialty-trained colorectal surgeons, a post-fellowship trainee in colorectal surgery with the Colorectal Surgical Society of Australia and New Zealand (CSSANZ), a Royal Australasian College of Surgeons (RACS) general surgical trainee, and eight pre-vocational medical officers. 

## Case presentation

A 72-year-old Caucasian man was referred to our unit from a regional hospital with a finding of an intra-luminal foreign body on an otherwise unremarkable colonoscopy organized following a positive fecal occult blood test (FOBT). His past surgical history was significant for an open left inguinal hernia repair using a polypropylene mesh plug 19 years prior, complicated by chronic wound infection with subsequent washout. He had also previously undergone an open appendicectomy. He had no other significant past medical history, took no regular medications, and was a lifelong non-smoker. On examination, he had a normal body mass index, and his abdomen was soft and non-tender with no palpable masses or hernias. On presentation, he reported four months of mild crampy abdominal pain with associated tenesmus. He denied a recent change in bowel movements or haematochezia. 

**Figure 1:**
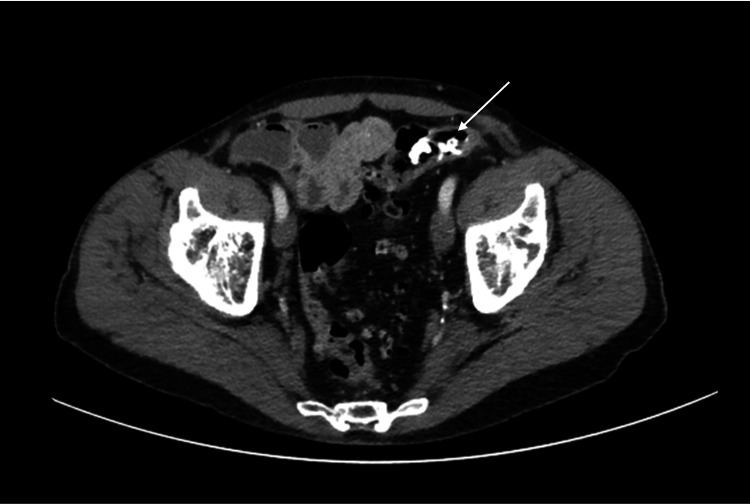
Axial CTAP showing calcified fragments at the descending/sigmoid colon junction (arrowed). CTAP = Computed tomography of abdomen and pelvis

**Figure 2:**
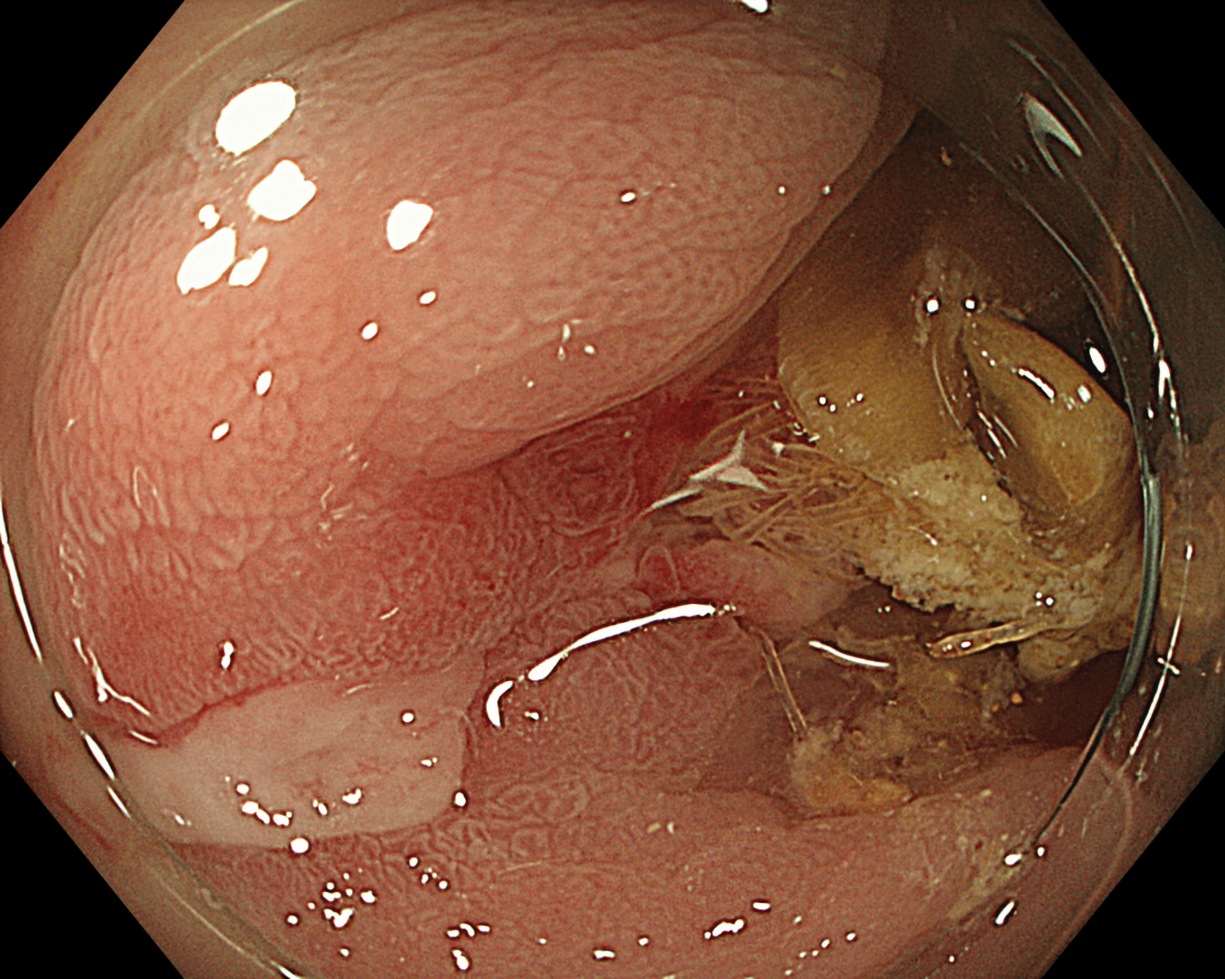
Endoscopic image of surgical mesh invading through colonic mucosa with adherent calcifications.

Routine laboratory investigations were normal (Table 1). Computed tomography of the abdomen and pelvis (CTAP) showed three nonspecific calcified fragments in the proximal sigmoid colon with associated diverticulosis (Figure 1). Flexible sigmoidoscopy confirmed the presence of surgical mesh invading through the colonic mucosa with associated granulation tissue and superficial ulceration (Figure 2). 

**Table 1:** Laboratory results on admission

Test	Result	Reference Range
White cell count	7.35	4.0-11.0 x 10^9^/L
Neutrophils	4.65	2-7.5 x 10^9^/L
Hemoglobin	138	135-180 g/L
Mean cell volume	90	80-100 fL
Creatinine	63	60-110 µL
Estimated glomerular filtration rate	>90	>60 ml/min/1.73 m^2^
C-reactive protein	<1	<5 mg/L

The patient subsequently underwent a laparoscopic high anterior resection. The procedure was performed by a post-fellowship trainee in colorectal surgery and a subspecialty-trained consultant colorectal surgeon. Intra-operatively, the sigmoid colon was adherent to the anterior abdominal wall, with a palpable mass of folded mesh (meshoma) present. The left inferior epigastric and gonadal arteries were involved in the meshoma and required ligation. The affected segment of colon was extracted via a Pfannenstiel incision, and an end-to-end stapled colorectal anastomosis was performed using a circular powered stapler. The patient's postoperative course was unremarkable, and he was discharged home on the sixth postoperative day. 

**Figure 3:**
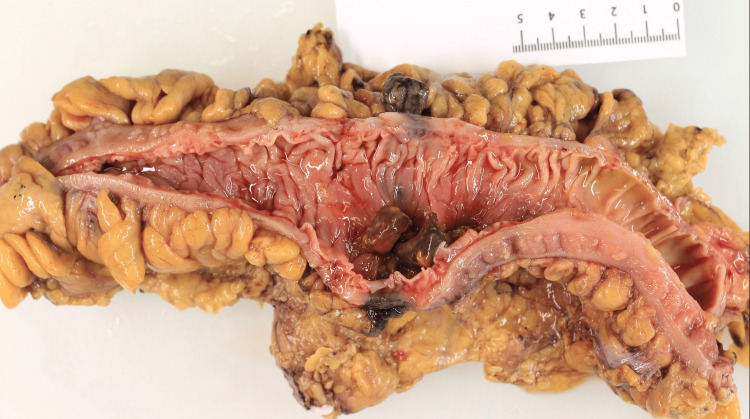
Pathological image of transected specimen with intraluminal mesh and calcified food fragments visible.

Final postoperative histopathology confirmed the findings of migrated hernia mesh attached to and invading through the colonic mucosa with associated intra-luminal calculi without evidence of dysplasia or malignancy (Figure 3).

## Discussion

This case highlights a rare potential complication of a routine surgical intervention. A summary of cases describing mesh migration involving the sigmoid colon following inguinal hernia repair since 1994 is listed in Table 2. This builds on the work of Gossetti et al., with an additional 9 such cases described since their comprehensive review published in 2018.

**Table 2:** Summary of published cases of mesh migration involving the sigmoid colon following inguinal hernia repair. TAPP = trans-abdominal preperitoneal; MP = mesh plug; TEP = totally extraperitoneal; IPOM = intraperitoneal onlay mesh; TIPP = transinguinal preperitoneal; PHS = prolene hernia system

Author	Latency period	Technique	Clinical Presentation	Treatment
Gray et al. [6]	N/A	TAPP	Colovesical fistula	Sigmoidectomy and suture repair
Hamy et al. [7]	2 years	Stoppa	Sigmoiditis	Hartmann’s
Hamy et al. [7]	9 months	Stoppa	Pelvic abscess	Sigmoidectomy
McDonald et al. [8]	N/A	TAPP	Large bowel obstruction	Sigmoidectomy
Weitzel et al. [9]	20 years	Lichtenstein	Colocutaneous fistula	Suture repair and ileostomy
Panzironi et al. [10]	5 years	TAPP	Large bowel obstruction	Sigmoidectomy
Tokunaga et al. [11]	7 years	MP	Rectal bleeding	Sigmoidectomy
Lauwers et al. [12]	2 years	Stoppa	Abdominal wall abscess	Sigmoidectomy
Bonner et al. [13]	2 years	TAPP	Rectal bleeding	Sigmoidectomy
Lange et al. [14]	N/A	TAPP	Rectal bleeding	Sigmoidectomy
Pautrat et al. [15]	5 years	Stoppa	Sigmoiditis	Sigmoidectomy
Benedetti et al. [16]	2 years	MP	Rectal bleeding	Hartmann’s
Murphy et al. [17]	2 years	MP	Left lower quadrant pain	Sigmoidectomy
Zubaidi et al. [18]	2 years	MP	Colocutaneous fistula	Left hemicolectomy
Benes et al. [19]	4 months	TAPP	Rectal bleeding	Suture repair
Benes et al. [19]	7 months	TAPP	Rectal bleeding	Suture repair
Benes et al. [19]	9 months	TAPP	Rectal bleeding	Sigmoidectomy
Kurukahvecioglu et al. [20]	1 year	IPOM	Bloody diarrhea	Removal of mesh
Barreto et al. [21]	4 years	TIPP	Colovesical fistula	Anterior resection and suture repair
Tamam et al. [22]	7 years	TEP	Colovesical fistula	Sigmoidectomy and suture repair
Ishiguro et al. [23]	3 years	MP	Colocutaneous fistula	Sigmoidectomy
El Hakam et al. [24]	7 years	Lichtenstein	Hematochezia	Sigmoidectomy
Szitkar et al. [25]	7 months	N/A	Sigmoiditis	Left hemicolectomy
Karls et al. [26]	12 years	TAPP	Sigmoiditis	Sigmoidectomy
Zuvela et al. [27]	6 years	PHS	Cutaneous fistula	Suture repair
Yilmaz et al. [28]	3 years	MP	Acute abdomen	Sigmoidectomy
Ratajczak et al. [29]	2 years	MP	Left lower quadrant mass	Sigmoidectomy
Al-Subaie et al. [30]	3 years	Lichtenstein	Rectal bleeding	Sigmoidectomy
Tajima et al. [31]	10 years	MP	Colocutaneous fistula	Sigmoidectomy
Rieger et al. [32]	N/A	TAPP	Colovesical fistula	N/A
Degheili et al. [33]	5 months	TAPP	Scrotal fistula	Laparotomy and suture repair
Unlisted Authors [34]	5 years	TAPP	Colocutaneous fistula	Sigmoidectomy
Scaringi et al. [35]	26 years	MP	Colocutaneous fistula	Sigmoidectomy
Han et al. [36]	2 months	TEP	Pelvic abscess	Sigmoidectomy
Chan et al. [37]	12 years	Lichtenstein	Diarrhea	Sigmoidectomy
Na et al. [38]	4 years	TAPP	Hematochezia	Sigmoidectomy
Ramanathan et al. [39]	N/A	N/A	Colovesical fistula	Suture repair
Liu et al. [40]	7 years	Stoppa	Sigmoiditis	Sigmoidectomy
Patel et al. [2]	10 years	TEP	Left lower quadrant pain	Hartmann’s
Fajardo et al. [3]	4 years	TAPP	Acute abdomen	Sigmoidectomy
Křístek et al. [41]	15 years	TAPP	FOBT +ve	Sigmoidectomy
Gang et al. [42]	8 years	TEP	FOBT +ve	Sigmoidectomy
Behbehani et al. [43]	7 years	TAPP	Left lower quadrant pain	Sigmoidectomy
Cardoso et al. [44]	3 years	TAPP	Generalized abdominal discomfort	Sigmoidectomy
Koliakos et al. [45]	20 years	Lichtenstein	Left lower quadrant pain	Hartmann’s
Sasaki et al. [46]	19 years	MP	Asymptomatic	Hartmann’s

Although a similar case has been recently reported in an asymptomatic patient [46], most patients with mesh migration into the colon present with nonspecific lower gastrointestinal symptomatology across a wide spectrum, including both fresh and occult rectal bleeding, as seen in this case. Time to presentation also exists across a wide spectrum, with cases describing presentation from as little as two months [36] to 26 years [35] post-hernia repair. Further study to evaluate whether certain techniques for hernia repair have a higher propensity to migrate, and at what stage, is required, with each of the most common techniques represented in Table 2. 

This case also highlights potential risk factors for migration, including sigmoid diverticulosis and postoperative wound infection associated with mesh migration. The presence of sigmoid diverticulosis may be a contributing factor in the development of sigmoidal involvement, particularly in left-sided inguinal hernia repair. Additionally, factors that increase the risk of postoperative infection, such as smoking status, diabetes, and immunosuppression, should be considered when consenting and planning left-sided inguinal hernia repair in such patients [47].

Additional considerations should be given to the type of mesh used during inguinal hernia repair, particularly in patients with risk factors as mentioned above. A number of similar cases reported have occurred in those patients who had hernia repairs using an open mesh plug, as in this case. Although this technique has fallen out of fashion of late [48], such mesh plugs are utilized frequently in laparoscopic hernia repair, with some arguing for their discontinuation [49], which may be a factor for the higher incidence of mesh migration complications amongst reported cases, although a formal comparative study is still required. 

Presentation due to mesh migration following inguinal hernia repair may become more common in the years ahead [50], as those who had such hernias repaired with mesh plugs during their initial popularity develop symptoms and late complications.

## Conclusions

Mesh migration into the sigmoid colon remains a rare complication of left-sided inguinal hernia repair. Consideration should be given to this diagnosis in those with risk factors for migration, such as diverticulosis, infection, and hernia repair using a mesh plug. Mesh migration into the sigmoid colon may become more common in the years ahead as those who underwent open left-sided inguinal hernia repair with such mesh plugs develop this late complication. 
